# Patterns of Delaying Surgery for Breast Cancer During the COVID-19 Outbreak in Daegu, South Korea

**DOI:** 10.3389/fsurg.2020.576196

**Published:** 2020-10-08

**Authors:** Jeeyeon Lee, Jin Hyang Jung, Wan Wook Kim, Chan Sub Park, Ho Yong Park

**Affiliations:** Department of Surgery, School of Medicine, Kyungpook National University, Kyungpook National University Chilgok Hospital, Daegu, South Korea

**Keywords:** COVID-19, breast carcinoma, surgery, delay, Korea

## Abstract

**Background:** The coronavirus disease 2019 (COVID-19) outbreak in South Korea has affected the diagnosis, treatment, and follow-up protocols of various cancers. This study investigated the patterns of delaying surgery for breast cancer during the COVID-19 outbreak in South Korea and evaluated factors that may have affected the decision to delay surgery.

**Methods:** From February 18 to April 18, 2020, which was the critical period for COVID-19 in South Korea, patients with breast cancer who were scheduled for surgery were evaluated in terms of their decision in delaying the procedure. The patients were divided into two groups: delaying and non-delaying surgery groups. The association between personal and clinicopathological factors and delaying surgery was evaluated.

**Results:** In patients belonging to the delaying surgery group, the mean delay period was 15.9 (standard deviation [SD], ±10.9) days. Patients in the non-delaying surgery group were relatively younger (*p* = 0.003), single (*p* = 0.038), had planned mastectomy (*p* = 0.041), received needle biopsy for diagnosis (*p* = 0.021), and had a higher clinical N stage (*p* = 0.049) and multifocal lesions of breast cancer (*p* = 0.020). However, there were no significant differences in terms of the pathological T and N stages between the two groups.

**Conclusion:** During the COVID-19 outbreak, there was no occurrence of nosocomial infection in the non-delaying surgery group and no statistical difference in pathological stage between the delaying and non-delaying surgery groups. Although patients in the delaying surgery group tended to be relatively older and married and had planned small-scale surgery with a good prognosis of breast cancer, the prognosis did not appear to have changed whether delaying or proceeding with surgery for breast cancer during the COVID-19 outbreak.

## Introduction

As of February 17, 2020, only 30 cases of coronavirus disease 2019 (COVID-19) had been reported in South Korea. However, on February 18, 2020, the 31st patient with confirmed COVID-19 was reported in Daegu, the third largest city in South Korea. COVID-19 has rapidly spread due to this patient's worships in the “Shincheonji” religious group (1, 2). As of May 2020, >10,000 COVID-19 cases had been confirmed, and the situation is still ongoing; hence, people are observing social distancing (3).

The COVID-19 outbreak has caused delays in the diagnosis and treatment of patients with cancer worldwide. Several oncology groups have established cancer guidelines during the COVID-19 pandemic, and physicians have managed such patients by following these guidelines (4–6). In South Korea, the most severe outbreak occurred in Daegu, and fatal medical conditions progressed on a daily basis (1, 7–10). Two Kyungpook National University Hospitals, the only national hospitals in the city, began accepting patients with COVID-19, thereby introducing the risk of infection among patients with cancer during their hospital stay.

The present study investigated the patterns of delaying surgery for breast cancer during the COVID-19 outbreak and evaluated the clinical outcomes between patients with breast cancer who postponed surgery and those who proceeded with surgery.

## Methods

A total of 94 newly diagnosed patients with breast cancer visited the Surgery Department of Kyungpook National University Chilgok Hospital from February 18 to April 18, 2020. They were subjected to initial treatment, including surgery, neoadjuvant chemotherapy, or neoadjuvant radiotherapy. Among them, 62 patients with breast cancer were determined for surgery first; three surgical oncologists planned to perform surgeries for these patients.

Written informed consent was obtained from all patients, and the protocol used in this study was approved by the Institutional Review Board (IRB) Committee of Kyungpook National University Chilgok Hospital, Daegu, Republic of Korea (KNUCH 2020-03-009). The specific inclusion and exclusion criteria were defined in the approved IRB protocol. The data were collected and analyzed retrospectively.

On the first day of the COVID-19 outbreak (February 18, 2020), the surgeons made brief phone calls to enquire whether patients wanted to postpone the surgery. Most patients made decisions within 2–3 days after discussion with their families. One patient who wanted to advance the time of surgery was excluded from this study.

The emergency response team from Kyungpook National University Chilgok Hospital established a guideline for elective surgery as follows: (1) every patient planning to have elective surgery should undergo the routine COVID-19 test 2–3 days prior to surgery; (2) the COVID-19 test can be conducted at 9:00 a.m. (results by 1:30 p.m.) or at 3:00 p.m. (results by 7:00 p.m.) at a drive-through screening center; and (3) the elective surgery can only be permitted once the COVID-19 results are negative, and one isolated negative-pressure surgery room would be designated for an emergency surgery of a patient with COVID-19 (11–13). However, most of the conventional protocols for various cancers were maintained during the COVID-19 outbreak.

The initial and final operation days were retrospectively investigated after the surgery was completed. The patients were divided into two groups: delayed surgery group and non-delayed surgery group. Their personal history and clinicopathological factors were reviewed, and the association between delaying surgery and clinicopathological factors was determined.

## Statistical Analysis

Statistical analysis was performed using SPSS version 25.0 (SPSS, Chicago, IL, USA), and categorical variables were analyzed using the chi-squared test. *P*-values of < 0.05 were considered statistically significant.

## Results

Among the 62 patients with breast cancer, 27 (SD, ±43.5%) postponed surgery during the COVID-19 outbreak. The mean age of all the patients was 55.7 ± 11.2 years, and the cohort comprised only one male patient with breast cancer. Among the patients, 36 (58.1%) were postmenopausal women and 8 (12.9%) were single (unmarried or divorced). More than 88% of the patients had offspring, and the mean number of offspring was two (SD, ±0.9) (Table 1).

**Table 1:** Characteristics of patients who underwent surgery during the COVID-19 outbreak.

	**Total patients (*n* = 62)**	**Delayed surgery group (*n* = 27)**	**Non-delayed surgery group (*n* = 35)**	***p*-value**
Mean age (years, ± SD)	55.7 ± 11.2	58.4 ± 11.9	55.2 ± 10.8	0.003
>50 years				
<50 years				
Gender (F:M)	61:1	27:0	34:1	
Mean body mass index (kg/m^2^, ± SD)	42.0 ± 3.7	24.2 ± 3.9	23.9 ± 3.5	0.741
Mean menarche age (years, ± SD)	15.1 ± 0.8	15.3 ± 1.7	14.9 ± 1.9	0.429
Mean menstruation cycle (days, ± SD)	28.6 ± 1.8	28.1 ± 1.9	29.0 ± 4.4	0.601
Menopausal status (n, %)				0.561
Premenopause	26 (41.9)	10 (37.0)	16 (45.7)	
Postmenopause	36 (58.1)	17 (63.0)	19 (54.3)	
Marriage status (*n*, %)				0.038
Unmarried	8 (12.9)	2 (7.4)	6 (17.1)	
Married	54 (87.1)	25 (92.6)	29 (82.9)	
Having offspring (*n*, %)	55 (88.7)	24 (88.9)	30 (85.7)	0.321
Mean number of offspring (± SD)	2.0 ± 0.9	2.0 ± 1.2	1.9 ± 1.0	
Family history of breast cancer (*n*, %)	6 (9.7)	0	6 (17.1)	
Type of breast surgery (*n*, %)				0.041
Breast conserving surgery	45 (72.6)	22 (81.5)	23 (65.7)	
Mastectomy	17 (27.4)	5 (18.5)	12 (34.3)	
Type of axillary surgery (*n*, %)				0.194
No surgery	2 (3.2)	2 (7.4)	0	
Sentinel lymph node biopsy	57 (91.9)	24 (88.9)	33 (94.3)	
Axillary lymph node dissection	3 (4.8)	1 (3.7)	2 (5.7)	
Immediate breast reconstruction (*n*, %)				0.104
Yes	11 (17.7)	2 (7.4)	9 (25.7)	
No	51 (82.3)	25 (92.6)	26 (74.3)	
Prior neoadjuvant chemotherapy (*n*, %)	2 (3.2)	1 (3.7)	1 (2.9)	
Adjuvant chemotherapy (*n*, %)	19 (30.6)	9 (33.3)	10 (28.6)	
Adjuvant radiotherapy (*n*, %)	44 (71.0)	20 (74.1)	24 (68.6)	
Adjuvant hormone treatment (*n*, %)	49 (79.0)	23 (85.2)	26 (74.3)	

For the diagnosis of breast cancer, 6 patients (9.7%) had undergone excision biopsy and 13 (21.0%) had ductal carcinoma *in situ*, and the mean clinicopathological tumor size in the patients was 2.1 (SD, ±1.6) and 1.9 (SD, ±1.6) cm, respectively. Only one patient underwent breast-conserving surgery without sentinel lymph-node biopsy (Table 2). There were no cases for which the surgical plan was changed during the COVID-19 outbreak.

**Table 2:** Disease characteristics of patients with breast cancer who underwent surgery during the COVID-19 outbreak.

	**Total patients (*n* = 62)**	**Delayed surgery group (*n* = 27)**	**Non-delayed surgery group (*n* = 35)**	***p*-value**
Multifocality (*n*, %)	11 (17.1)	3 (11.1)	8 (22.9)	0.020
Biopsy method (*n*, %)				0.021
Core needle biopsy	56 (90.3)	23 (85.2)	33 (94.3)	
Excision biopsy	6 (9.7)	4 (14.8)	2 (5.7)	
Type of breast cancer (*n*, %)				0.774
Non-invasive carcinoma	13 (21.0)	6 (22.2)	7 (20.0)	
Invasive carcinoma	49 (79.0)	21 (77.8)	28 (80.0)	
Histologic type of breast cancer (*n*, %)				
Ductal carcinoma *in situ*	13 (21.0)	6 (22.2)	7 (20.0)	
Invasive ductal carcinoma	45 (72.6)	19 (70.4)	26 (74.3)	
Invasive lobular carcinoma	3 (4.8)	2 (7.4)	1 (2.9)	
Mucinous cancer	1 (1.6)	0	1 (2.9)	
Mean clinical tumor size (cm, ± SD)	2.1 ± 1.6	2.0 ± 1.2	2.2 ± 1.8	0.912
Clinical T stage (*n*, %)				0.849
cT1	38 (61.3)	15 (55.6)	23 (65.7)	
cT2	10 (16.1)	6 (22.2)	4 (11.4)	
cT4	1 (1.6)	0	1 (2.9)	
Clinical N stage (*n*, %)				0.049
cN0	58 (93.5)	26 (96.3)	32 (91.4)	
cN1	3 (4.8)	1 (3.7)	2 (5.7)	
cN3	1 (1.6)	0	1 (2.9)	
Mean pathologic tumor size (cm, ± SD)	1.9 ± 1.6	1.8 ± 1.2	2.0 ± 1.8	0.817
Pathological T stage (*n*, %)				0.274
pTis	13 (21.0)	6 (22.2)	7 (20.0)	
pT1	34 (54.8)	15 (55.6)	19 (54.3)	
pT2	14 (22.6)	6 (22.2)	8 (22.9)	
pT4	1 (1.6)	0	1 (2.9)	
Pathological N stage^*^ (*n*, %)				0.658
pN0	43 (69.4)	17 (63.0)	26 (74.3)	
pN1	16 (25.8)	9 (33.3)	7 (20.0)	
pN3	2 (3.2)	0	2 (5.7)	

* *One patient had received only breast-conserving surgery without sentinel lymph node biopsy*.

**Figure 1:**
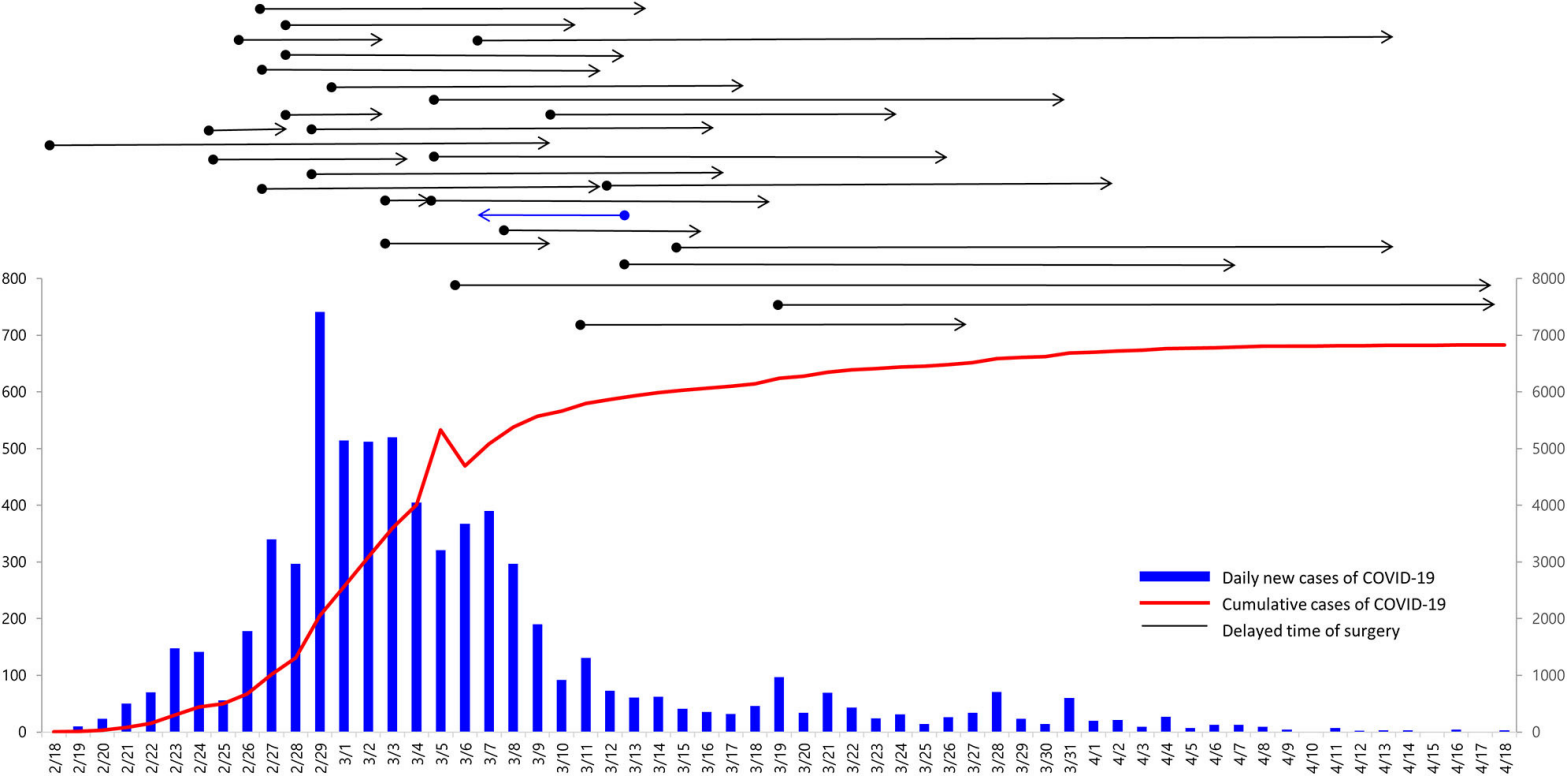
Daily and cumulative number of COVID-19 cases in Daegu and delayed time of surgery for breast cancer during the COVID-19 outbreak. Overall, 27 patients wished to delay surgery to avoid COVID-19 infection in the hospital (black arrow lines). One patient who was excluded from this study wished to have an advanced operation day (blue arrow line).

The COVID-19 outbreak in Daegu had worsened since February 18, 2020, and the cumulative number of COVID-19 cases increased to more than 6000 after March 15, 2020. However, only one or two patients per day were confirmed to have COVID-19 after April 11, 2020. During the COVID-19 outbreak in Daegu, the mean delay period of surgery was 15.9 (SD, ±10.9) days (Figure 1).

**Figure 2:**
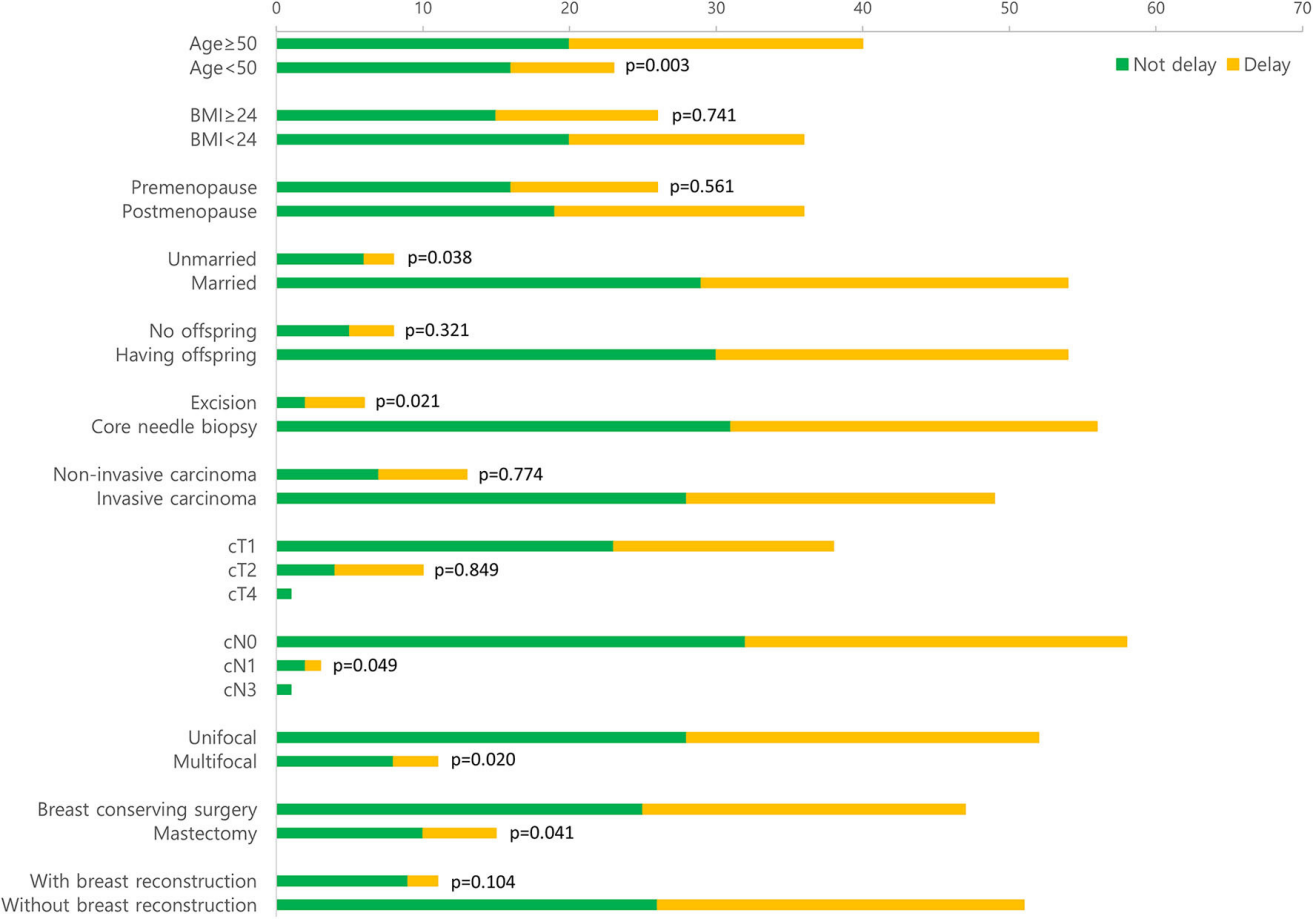
Comparison of personal and clinicopathological factors between the delaying and non-delaying surgery groups.

In the delayed surgery group, more than 5% of patients aged <50 years did not want to delay surgery, which was statistically significant (*p* = 0.003), and single patients (unmarried or divorced) tended to postpone the surgery (*p* = 0.038). Patients diagnosed with breast cancer via excision biopsy were likely to delay surgery compared with those diagnosed with breast cancer via core needle biopsy (*p* = 0.021). Although the clinical T stage was not associated with the delay of surgery for breast cancer, the clinical N stage was weakly associated with the delay of surgery (*p* = 0.049). Patients who had multifocal breast cancer or were due for mastectomy were inclined to pursuing surgery (*p* = 0.020 and 0.041, respectively) (Figure 2). However, there was no significant difference in the pathological T and N stages between the two groups (*p* = 0.274 and 0.658, respectively).

When comparing the amount of time surgery was delayed (≤ 1 week, >1 week, ≤ 1 month, and >1 month), only the clinical (same as pathological) T stage showed statistical significance among the three groups (*p* = 0.003). No other clinical and pathological variables were different among the three groups (Table 3).

**Table 3:** Clinicopathological characteristics of patients with breast cancer who delayed surgery during the COVID-19 outbreak.

**Delaying time**	**≤1 week (*n* = 7)**	**>1 week and ≤1 month (*n* = 18)**	**>1 month (*n* = 1)**	***p-*value**
Mean age (years, ± SD)	55.0 ± 13.2	56.7 ± 12.1	67.0	0.842
Mean body mass index (kg/m^2^, ± SD)	22.6 ± 3.0	24.7 ± 4.3	23.5	0.203
Postmenopausal status (*n*, %)	4 (57.2)	12 (66.7)	1 (100.0)	0.713
Marriage status (*n*, %)				0.648
Unmarried	0	2 (11.1)	0	
Married	7 (100.0)	16 (88.9)	1 (100.0)	
Having offspring (*n*, %)	6 (85.7)	16 (88.9)	1 (100.0)	0.921
Mean number of offspring (± SD)	1.6 ± 0.8	2.1 ± 1.4	3	
Type of breast surgery (*n*, %)				0.070
Breast conserving surgery	5 (71.4)	16 (88.9)	0	
Mastectomy	2 (28.6)	2 (11.1)	1 (100.0)	
Type of axillary surgery (*n*, %)				0.936
No surgery	1 (14.3)	1 (5.6)	0	
Sentinel lymph node biopsy	5 (71.4)	17 (94.5)	1 (100.0)	
Axillary lymph nodes dissection	1 (14.3)	0	0	
Immediate breast reconstruction (*n*, %)				0.356
Yes	1 (14.3)	2 (11.1)	0	
No	6 (85.7)	16 (88.9)	1 (100.0)	
Multifocality (*n*, %)	1 (14.3)	2 (11.1)	0	0.886
Axillary lymph node metastasis (*n*, %)	1 (14.3)	7 (38.9)	0	0.553
Clinical T stage^*^ (*n*, %)				0.003
cTis	4 (57.2)	1 (5.6)	1 (100.0)	
cT1	3 (42.9)	11 (61.1)	0	
cT2	0	6 (33.3)	0	
Clinical N stage^*^ (*n*, %)				0.750
cN0	5 (71.4)	11 (61.1)	1 (100.0)	
cN1	2 (28.6)	7 (38.9)	0	
Prior neoadjuvant chemotherapy (*n*, %)	1 (14.3)	0	0	0.211
Adjuvant chemotherapy (*n*, %)	2 (28.6)	7 (38.9)	0	0.719
Adjuvant radiotherapy (*n*, %)	5 (71.4)	16 (88.9)	0	0.073
Adjuvant hormone treatment (*n*, %)	4 (57.2)	17 (94.5)	1 (100.0)	0.231

* *Clinical T and N stages were the same as pathological T and N stages*.

## Discussion

There had been an unexplained lower respiratory infectious disease in Wuhan, Hubei Province, China, in November 2019, and the first case was reported on December 31, 2019, by the World Health Organization Country Office in China. This disease entity originated in a novel variant virus belonging to the coronavirus family and is thus named “COVID-19” (14).

Because China is an Asian country, the COVID-19 pandemic instantly spread to other adjacent countries, including South Korea and Japan. In South Korea, although only 30 cases had been reported as of February 17, 2020, the situation was worsened by the 31st patient who spread COVID-19 in Daegu (1, 7–10). Emergency medical conditions directly affect the diagnosis and treatment of cancer (15–18), particularly in the case of breast cancer. Because our institution, a national university hospital catering to patients with cancer in Daegu, had to accept patients with COVID-19 in the intensive care units and negative-pressure rooms, patients with cancer who were being treated at our hospital had experienced fear and panic. Many patients with breast cancer who were scheduled for surgery wanted to postpone the surgery until the COVID-19 situation subsided.

More than 40% of patients (26/62 patients) decided to delay the surgery, with a mean delay period of 15.9 days. Significantly, patients with breast cancer who were aged >50 years, were married, received excision biopsy, had planned breast-conserving surgery, and were having lower clinical N stage and unifocal cancer wanted to delay the surgery. Delayed surgery was generally preferred by patients with a lower stage of breast cancer, those with simple cases, and those who considered COVID-19 as a threat to their families.

Although the delay of surgery for breast cancer was not a result of only patients' desire but also took the overall condition in the hospital into consideration, there was a tendency to delay surgery for patients who were expected to have relatively better prognosis and who have family support.

During the COVID-19 outbreak in South Korea, several patients with breast cancer wanted to delay surgery until the COVID-19 pandemic could subside. The patients who delayed surgery were relatively older, were married, were due for breast-conserving surgery, had their breast cancer diagnosed via core needle biopsy, and had a higher clinical N stage or multifocality than those who opted to pursue surgery. However, there was no significant difference in terms of the pathological stage between the two groups, even if the surgery was delayed for several weeks.

In conclusion, whether the surgery for breast cancer was delayed or proceeded as planned, neither nosocomial infection of COVID-19 nor worsening of breast cancer occurred during the COVID-19 outbreak.

## Data Availability Statement

The raw data are available from the corresponding author on reasonable request.

## Ethics Statement

Written informed consent was obtained from all patients and the protocol used in this study was approved by the Institutional Review Board (IRB) Committee of Kyungpook National University Chilgok Hospital, Daegu, Republic of Korea (KNUCH 2020-03-009). The specific inclusion and exclusion criteria were defined in the approved IRB protocol. The data were collected and analyzed retrospectively.

## Author Contributions

JL and CSP contributed to the study design and manuscript writing. CSP, JHJ, WWK, and HYP conducted literature research and clinical practice. JHJ, WWK, and HYP also contributed to the data acquisition and analysis. JL reviewed the manuscript and made revisions. All authors read and approved the final manuscript. The figures were made by authors' own work.

## Conflict of Interest

The authors declare that the research was conducted in the absence of any commercial or financial relationships that could be construed as a potential conflict of interest.

## Abbreviations

COVID-19: coronavirus disease 2019.
